# Successful Management of Pharyngeal Perforation Caused by Overtube Insertion During Endoscopic Submucosal Dissection

**DOI:** 10.7759/cureus.8090

**Published:** 2020-05-13

**Authors:** Kazuya Inoki, Kenichi Konda, Atsushi Katagiri, Fuyuhiko Yamamura, Hitoshi Yoshida

**Affiliations:** 1 Department of Medicine, Division of Gastroenterology, Showa University School of Medicine, Tokyo, JPN

**Keywords:** overtube, endoscopy, pharynx, perforation, treatment, adverse event

## Abstract

A woman in her 70s underwent endoscopic submucosal dissection (ESD) for gastric-type adenoma in the anterior wall of the upper gastric body with intravenous anesthesia. We decided to use an overtube to control the air volume in the stomach. The overtube was inserted under endoscopic guidance using a sufficient amount of lubricating jelly. We encountered resistance when the top of the overtube was advanced to the pharynx; therefore, we stopped the overtube insertion and pulled the tube out immediately. We observed a linear injury in the posterior wall of the hypopharynx. The injury was deep and diagnosed as a pharyngeal perforation. Computed tomography (CT) revealed free air in the neck, with mediastinal emphysema. Conservative treatment was initiated after consultation with the otorhinolaryngologist; the patient received nothing per mouth and was administered intravenous antibiotics. The patient did not develop a fever and no signs of inflammation were observed. CT performed on postoperative day (POD) 5 revealed the disappearance of the mediastinal emphysema and a soft diet was introduced. The patient was discharged on POD 7. The ESD was postponed to two months later and was performed successfully. The scar of the perforation site was confirmed. In this report, we describe an extremely rare adverse event associated with overtube insertion. Although the incidence of pharyngeal perforation is low and its management is controversial, it was done without surgical intervention in the present case.

## Introduction

A flexible overtube is occasionally used during endoscopic procedures, such as endoscopic submucosal dissection (ESD), to achieve hemostasis in patients with ruptured esophageal varices or foreign body removal [1-4]. Although rare, overtube-induced adverse events are known to occur [5-8]. They include pharyngeal or esophageal perforations, vocal cord paralysis, and ruptured varices. Of these, the optimal management of pharyngeal perforation is controversial; additionally, its clinical course has not been fully described so far. In this study, we report the perforation of the posterior hypopharyngeal wall secondary to overtube insertion in a patient who responded to conservative treatment.

## Case presentation

**Figure 1:**
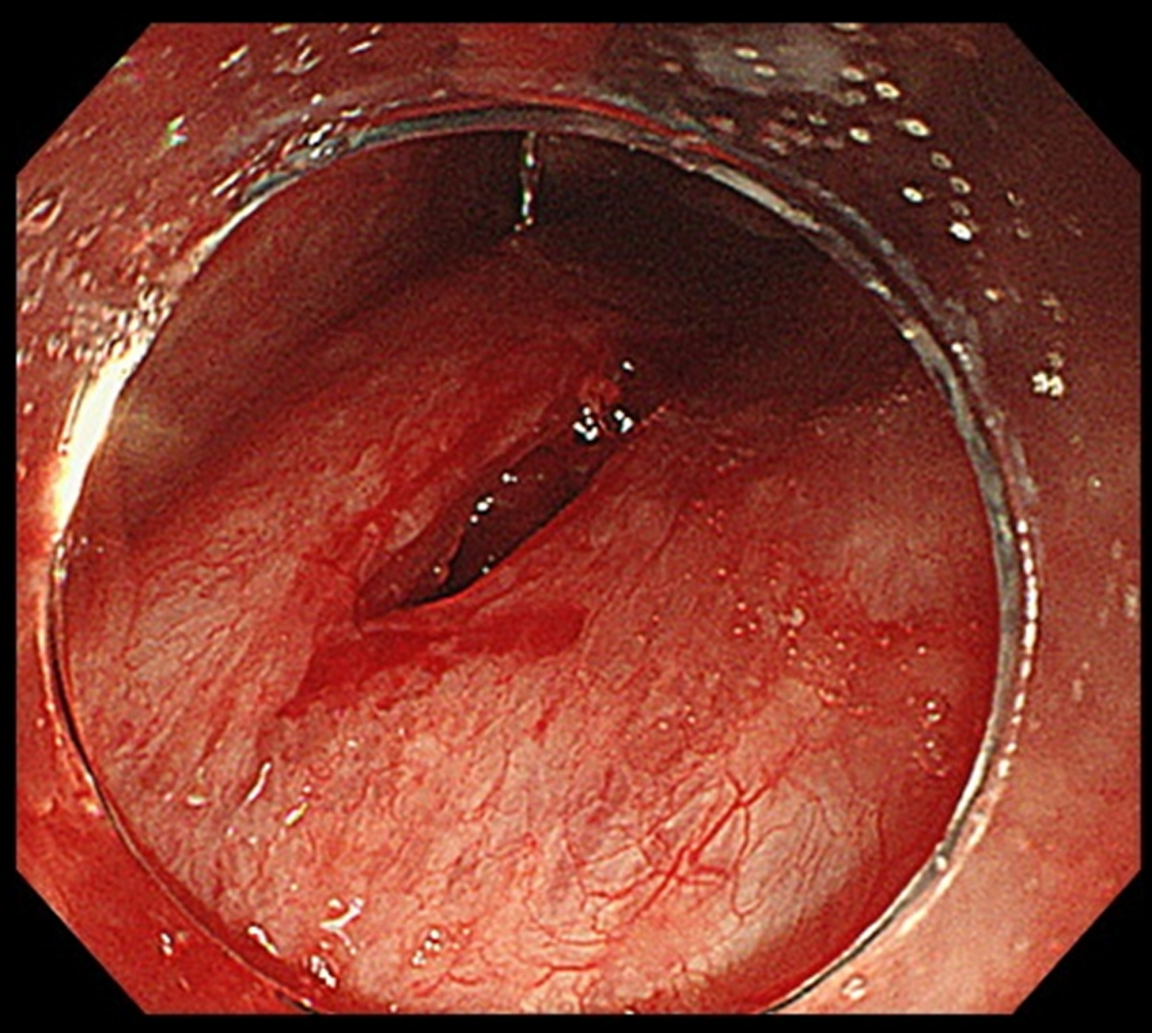
Overtube-induced laceration in the posterior wall of the oropharynx

A woman in her 70s had been followed for atrophic gastritis by annual esophagogastroduodenoscopy. A gastric-type adenoma in the anterior wall of the upper gastric body was detected and ESD was performed with intravenous anesthesia. Adequate air insufflation was not possible during gastric ESD owing to severe belching; therefore, we decided to use an overtube (Flexible Overtube®; Sumitomo Bakelite, Tokyo, Japan) to control the air volume in the stomach. The overtube was inserted under endoscopic guidance using a sufficient amount of lubricating jelly with the longer tip toward the anterior wall side. We encountered small resistance when the top of the overtube was advanced to the pharynx. Then, we swung the overtube slightly, however, the resistance remained; therefore, we stopped the overtube insertion and pulled the tube out immediately. We observed a linear injury in the posterior wall of the hypopharynx. The injury was deep and a space behind the posterior wall of hypopharynx was seen, therefore, it was diagnosed as a pharyngeal perforation (Figure 1).

**Figure 2:**
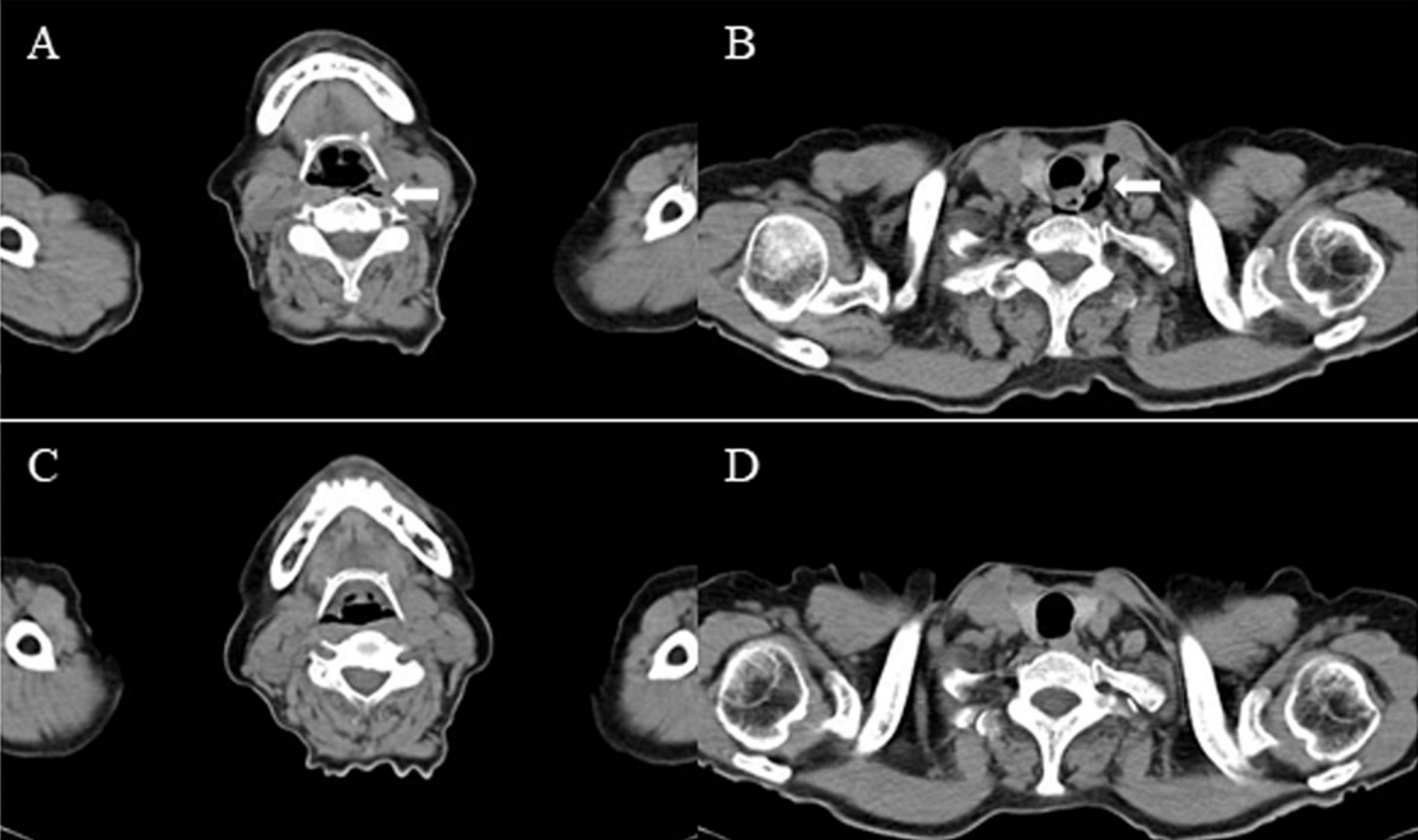
CT scan obtained immediately after ESD and follow-up CT scan obtained on postoperative day 5 CT scan obtained immediately after ESD (A) free air below the posterior wall of the oropharynx; (B) free air in the anterior mediastinum; (C) & (D) Follow-up CT scan obtained on postoperative day 5 showing the disappearance of the free air CT: computed tomography; ESD: endoscopic submucosal dissection

First, as with the case of gastrointestinal perforation during endoscopic treatment, we performed immediate closure with several endoclips (HX-610-135^Ⓡ^, Olympus Medical Systems Corp., Tokyo, Japan) to the site of the pharyngeal perforation. However, the patient started to suffer from its stimulus due to the contact of the clip with the epiglottis. Then, we removed all the clips. Computed tomography (CT) revealed free air in the neck with mediastinal emphysema (Figures 2A-2B). Conservative treatment with watchful waiting was initiated after consultation with the otorhinolaryngologist; the patient received nothing per mouth and was administered intravenous tazobactam/piperacillin (TAZ/PIPC) 4.5 g three times a day. Although the patient had a sore throat, she did not develop a fever and the symptom gradually improved. Further, no significant elevation of white blood cell (WBC) count and C-reactive protein (CRP) were observed on postoperative day (POD) 1 (WBC 6400/μl, CRP 0.21 mg/dL). She started to drink on POD 1. The intravenous TAZ/PIPC was switched to oral amoxicillin/clavulanate on POD 4, and amoxicillin/clavulanate was continued for 10 days. CT performed on POD 5 revealed the disappearance of the mediastinal emphysema (Figures 2C-2D).

**Figure 3:**
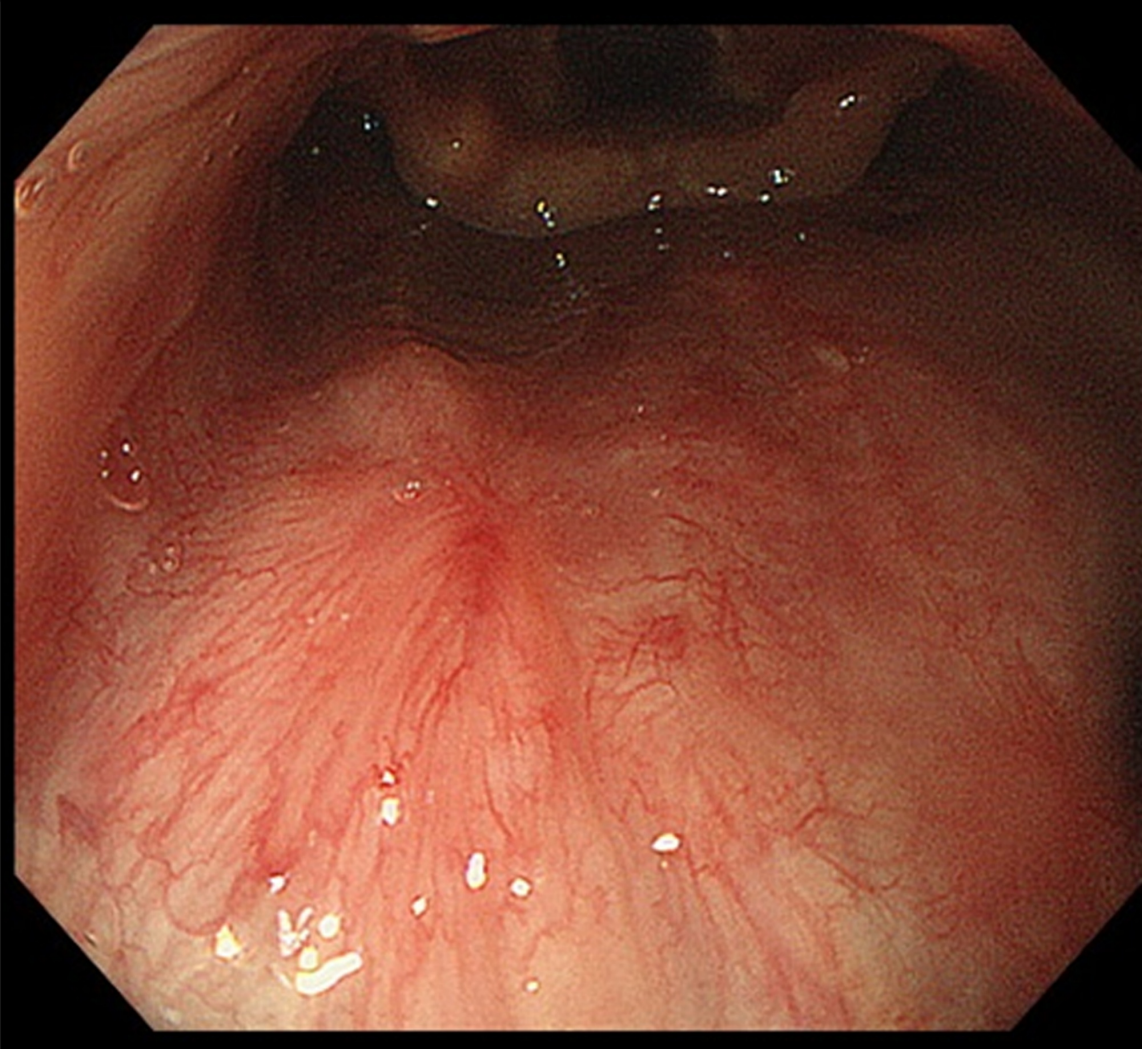
Healing scar in the posterior wall of the oropharynx

Then, a soft diet was introduced. The otorhinolaryngologist followed up on the perforation site and mucosal healing was confirmed on PODs 4 and 6. The patient was discharged on POD 7. The ESD was postponed to two months later and was performed successfully. The perforation was completely healed and the scar was confirmed endoscopically (Figure 3).

## Discussion

In this report, we describe an extremely rare adverse event associated with overtube insertion. When inserting the overtube, it is important to keep the longer tip toward the anterior wall side to avoid injury to the pharynx and esophagus. Further, it is also important to become sensitive to resistance. In the present case, there is the possibility that we swung the overtube too strongly, and, eventually, a pinch injury occurred. Pharyngeal perforation could occur due to several causes such as overtube insertion like in the present case, gastrointestinal endoscopy, transesophageal echocardiography, endotracheal intubation, nasogastric tube placement, and blunt trauma [9-14]. Further, ESD for pharyngeal cancers may carry a risk of pharyngeal perforation. Although the incidence of pharyngeal perforation is low and its management is controversial, it could be done without surgical intervention in the present case. Although rare, as endoscopists may encounter a pharyngeal perforation, it is essential to understand the anatomy of the neck area and possible management procedures to control pharyngeal perforation.

Due to the extremely low incidence of pharyngeal perforation, it was difficult for us to immediately resort to an appropriate strategy for pharyngeal perforation in this case. Although we performed clip closure initially, it failed due to severe stimulus for the patient. Endoscopic clip closure is effective for perforations of luminal organs; however, clip closure of the perforation of the pharyngeal area is inappropriate, as it causes stimulus, irritation, and possible dislodgment in the airway. Berkelhammer et al. reported a similar case of a patient who underwent surgical exploration via the supraclavicular approach for perforation site closure [6]. Further, Wasano et al. reported the successful transoral closure of a pharyngeal perforation due to gastrointestinal endoscope insertion [9]. Recently, a flexible endoscopic suturing system (ESS) and endoscopic hand suturing (EHS) have been developed [15-17]. If the maneuverability of ESS and EHS are improved, it would be a treatment option for pharyngeal perforation to achieve immediate closure in the future. In the present case, conservative treatment without surgical intervention, after consulting the otorhinolaryngologist, was provided. Unlike the perforation during ESD, there was no thermal effect at the perforation in the present case; consequently, there was a possibility that mucosal healing of the perforation of the present case would be better than that of ESD. Although consultation with the otorhinolaryngologist and close monitoring, along with administrating intravenous antibiotics to avoid life-threatening mediastinitis, is essential, it is possible for conservative treatment without surgical intervention to be an option for oropharyngeal perforation caused by an overtube. If patients did not respond to conservative treatment, prompt surgical intervention should be carried out.

Even though the pharynx is navigated during esophagogastroduodenoscopy, not many endoscopists are familiar with the anatomy of the neck area. Notably, behind the oropharyngeal wall, there is a retropharyngeal space (RFS), which extends from the clivus to the superior mediastinum [18]. The RFS is bounded by the buccopharyngeal fascia anteriorly, the alar fascia posteriorly, and the carotid sheath laterally. In the present case, the tip of the overtube broke the buccopharyngeal fascia and penetrated the retropharyngeal space.

## Conclusions

The present case showed a rare complication of the use of an overtube and is highly suggestive in that conservative treatment could be performed in selected cases even though mediastinum emphysema was present due to iatrogenic pharyngeal perforation.
